# Synthesis, Radioiodination and Biodistribution Evaluation of 5-(2-amimo-4-styryl pyrimidine-4-yl)-4-methoxybenzofuran-6-ol

**DOI:** 10.7508/aojnmb.2013.01.007

**Published:** 2013

**Authors:** Atteyat A. Labib

**Affiliations:** Inshas Cyclotron Facility, Nuclear Research Center, Atomic Energy Authority, Cairo 13759, Egypt

**Keywords:** Benzofuran derivatives, Iodine -125, imaging agents, Tissues distribution

## Abstract

This study describes the organic synthesis of 5-(2-amimo-4-styryl pyrimidine-4-yl)-4-methoxy benzofuran-6-ol (SPBF) as an example of a benzofuran derivative used as a new series of amyloid imaging agents. These benzofuran derivatives may be useful amyloid imaging agents for detecting B-amyloid plagues in the brain of Alzheimer’s disease. The precursor is 1-[6-hydroxy-4-methoxybenzofuran-5-yl]-phenyl butadiene ketone, which react with guanidine hydrochloride. The purification process was done via crystallization using solvent ethanol. The overall yield was 75% and the structure of the synthesized compound was confirmed by correct analytical and spectral data. Also, The synthesized compound was labeled with radioactive iodine -125 via electrophilic substitution reaction, in the presence of iodogen as an oxidizing agent, the labeling process was carried out at 95°C for 20min. The radiochemical yield was determined by using a thin layer chromatography and the yield was equal to 80%. Preliminary an in-vivo study examined normal mice after intravenous injection through the tail vein and the data showed the labeling compound was quickly cleared from most body organs. The radioiodinated compound showed high brain uptake. The results of this study suggest that radioiodinated (SPBF) may be useful as a brain imaging agents.

## INTRODUCTION

During the past decades, compounds bearing nitrogen containing heterocyclic rings have received much attention due to their increased chemotherapeutic value and brain penetrating abilities for the development of novel antimicrobials, anthelmintic (1), anti-depressant and agents used in the diagnosis of Alzheimer’s disease (2). Thus, pyridine derivatives continue to attract great interest due to the wide variety of interesting biological activities observed for these compounds, such as anticancer, analgesic, antimicrobial and antidepressant activities (3-5). Heterocyclic molecules can act as highly functionalized scaffolds and are known pharmacophores of a number of biologically active and medicinally useful molecules (6, 7). Cytotoxic drugs remain the mainstay of cancer chemotherapy and are administered with novel ways of therapy such as signal inhibitors (8). It is therefore important to discover novel cytotoxic agents with spectra of activity and toxicity that differ from current agents (8). Also the cytotoxic activity of some benzofuran derivatives were evaluated against HEPG2 (human liver carcinoma cell line) in comparison with 5-fluorouracil (9). Benzofuran derivatives possess a wide range of biological activities. They have been reported to possess antimicrobial (10-14), antitumor (13, 15-17), anti-inflammatory (15) activity etc. Benzothiazoles play a significant role as antibacterial (13, 15-17) and antifungal agents and it has been known that the benzofuran ring system incorporated with different heterocyclic moieties has a wide spectrum of anticancer activity against different types of carcinomas (18-23). There are also some derivatives of pyrazole used as receptors that have important biological effects like tetrahydro and tetrahydrocannabinol (THC), that exert many of their effects on the brain cannabinoid CB1 receptor. A ligand labeled with a radionuclide suitable for positron emission tomographic (PET) or single photon emission computed tomographic (SPECT) imaging could be used to examine the distribution of cannabinoid receptors in the living human brain. However, previous attempts to study THC, in which tetrahydro cannabinol was labeled in the omega position of the alkyl side chain, was only partially successful, probably because of a combination of high lipophilicity and low affinity (24, 25). In this study a simple method is described to prepare ^125^I-ASPMBF by direct iodination of SPBF with Auger-electron emitter iodine-125 using several oxidizing agents and the optimum conditions required to produce high labeling yield. Preliminary, an in vivo study of ^125^I-ASPMBF in normal mice was done to elucidate the biological behavior of this labeled compound.

## Materials and Methods

All melting points were uncorrected and in degree Celsius (MPA100 melting point Apparatus). The IR spectra were recorded on a pyeunicam sp-11100 spectrophotometer. Mass spectras were performed by a shimadzu Gc-MS-QP 100 Ex (shimadzu, Japan). Elemental analysis was carried out by the Micro analytical Research Center, Faculty of Science, Cairo University. Radioactivity was measured by the means of a gamma counter (Nucleus Model 2010) connected with a well type NaI (Tl) crystal. All other chemicals were purchased from Merck Co. Radioactive iodine-125 was purchased from the Institute of Isotopes Co. Ltd. (IZOTOP) Budapest, Hungary.

### Procedures

1-[6-hydroxy-4-methoxy benzofuran-5-yl]-5-phenylpenta-2,4-dien-1-one(II)

To a solution of compound (**I**) (3.2 gm, 0.01 mol) and cinamaldehyde (0.01 mol) in ethanol (30 ml) 10% alcoholic sodium hydroxide (5 ml) was added and the reaction mixture was stirred at room temperature for 30 min. The reaction mixture was acidified with hydrochloric acid and the resulting solid was washed with water and crystallized by ethanol to give compound (**II**) (Table 1).

**Table 1:**
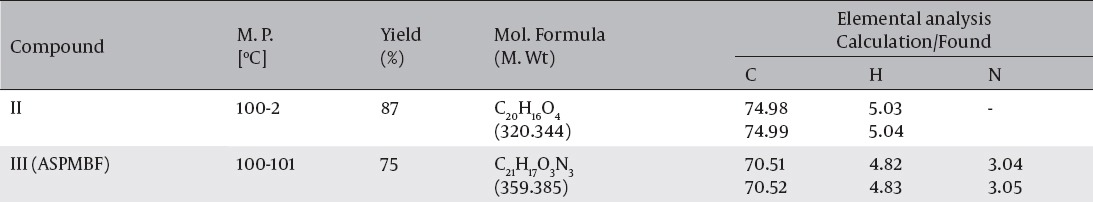
Characteristics data for the prepared compounds

5-(2-amimo-4-styryl pyrimidine-4-yl)-4-methoxy benzofuran-6-ol

A mixture of compound (**II**) (3.2 gm, 0.01 mol) guanidine hydrochloride (0.59 gm, 0.01 mol) and potassium hydroxide (0.5 gm) in ethanol (50 ml) was refluxed for 4 hours, then allowed to cool. The solid product was collected and crystallized from ethanol to produce compound (**III**).

### Iodination

The iodination process was achieved using two oxidizing agents, chloramine-T and iodogen. The ASPMBF compound was labeled with ^125^I using the chloramine–T method. Briefly, 0.2 mg of the compound was dissolved in 80µl of glacial acetic acid and to this solution 10µl of sodium ^125^I iodide (50 µCi) was added, followed by 20 µl of 0.1% chloramine-T solution in 0.05 M phosphate buffer (pH 7.4). After 2 min, the reaction mixture was quenched with 20 µl of 0.2% sodium metabisulfite in 0.05 M phosphate buffer (7.4). After adding 50 µl of saturated sodium bicarbonate, the radiochemical purity of labeled compound was checked by TLC, paper chromatographic methods and paper electrophoresis.

### Preparing iodogen coated tubes and coated glass frit

20 µl of iodogen (1 mM, 668.10^-6^ g/l) was dissolved in chloroform and transferred to glass tubes. CHCl_3_ was evaporated by dry N_2_ gas and iodogen was deposited on the wall of the glass tube as a thin film. The other method was carried out by dissolving 334×10^-6^ g/l of iodogen in a glass tube containing glass frit and chloroform; this system was then allowed to dry under dry N_2_ gas. These tubes were stored at 0°C until use.

### Determination of radiochemical yield and purity

Radiochemical yield and purity of the radiodinated compound was determined by

#### TLC chromatographic method

This technique was done using a thin layer silica gel coated on an aluminum sheet (20 cm×20 cm). It was cut into strips, each strip was 1.5 cm wide and 13 cm long; the spotted point was placed 2 cm above the edge. The solvent used for development was a methylene chloride : hexane mixture (4:1, v/v), radioiodide ^125^I remained near the origin (Rf=0-0.1), while the labeled compounds (^125^I-SPBF) moved to the solvent front ((Rf=0.9). the radiochemical yield (%) at the time (t), were calculated as the percent ratio of activity on the TLC-strip according to the following equation: Radiochemical yield (%)= Activity of labeled product×100 / total activity.

#### Paper chromatographic method

This technique was done using strips of Whatman paper. On a 1 cm wide and 13 cm long strip, 1-2 µl of the reaction mixture was placed 2 cm above the lower edge and allowed to evaporate spontaneously. For development, a fresh mixture of chloroform: ethanol (9:1, v/v) was used. After complete development, the paper sheet was removed, dried, and cut into strips, each strip was 1 cm wide, strips were then counted in a well type gamma counter.

#### Paper electrophoresis

Radiochemical yield was further confirmed by paper electrophoresis. On a Whatman paper sheet (2cm width and 47 cm length), 1-2 µl of the reaction mixture was placed 12cm above the lower edge and allowed to evaporate spontaneously. Electrophoresis was carried out for 1 h at a voltage of 300 V using normal saline (0.9% w/v NaCl solution) as an electrolytes source. After complete development, the paper was removed, dried, and cut into strips, each strip was 1 cm wide, and the strip were counted in a well type gamma counter. The percentage of radiochemical yield was estimated as the ratio of the radioactivity of radioiodinated compounds to the total activity multiplied by 100.

### Octanol distribution

Synthesized compound was mixed with a 1:1 (wt /wt) mixture of 1-octanol and 0.1 M phosphate buffer (pH 7.4), in a centrifuge tube. The mixture was vortexed at room temperature for 1 min and then centrifuged at 5,000 rpm for 5 min. Subsequently 100 µl samples from the 1-octanol and aqueous phases were pipetted into other test tubes and counted in a gamma counter. The measurement was repeated three times. The partition coefficient (P) was calculated as the ratio of optical density in the organic phase to the optical density in the aqueous phase (26-28).

### Biodistribution studies

#### Animals

Albino type mice, weighing 20-25g were used for the biological distribution study.

#### Method

This experiment was done by diluting the neutral solution of labeled ^125^I-ASPMBF with 3ml of saline for injection and the resulting solution was filtered through a 0.22 µm Millipore filter into a sterile sealed vial.100 µl (100-150 MBq) was injected in the tail vein of Albino white mice (3 groups each with 3 mice, with approximate weight of 25 g). The mice were maintained on a normal diet in metabolic cage. The mice were sacrificed at 30min and 1 h post- injection. Samples of fresh blood, bone and muscle were collected in pre-weighted vials and counted. The different organs were removed, counted and compared to a standard solution of the ^125^I-ASPMBF.

### The in-vitro stability

The stability assessment of the labeled product was performed by TLC, using a methylene chloride : hexane mixture (4:1, v/v) as a developing solvent. The study continued for up to 36 h and the data was recorded at pre-planned time intervals.

## RESULTS AND DISCUSSION

### Chemical synthesis

**Scheme 1:**
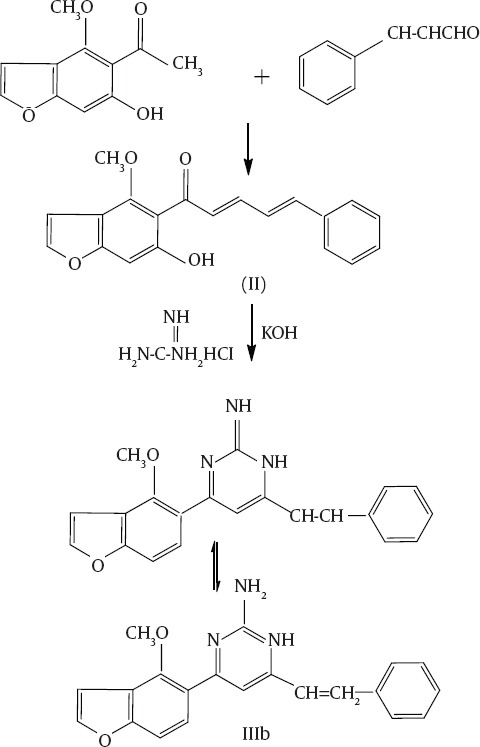


**Chart 1:**
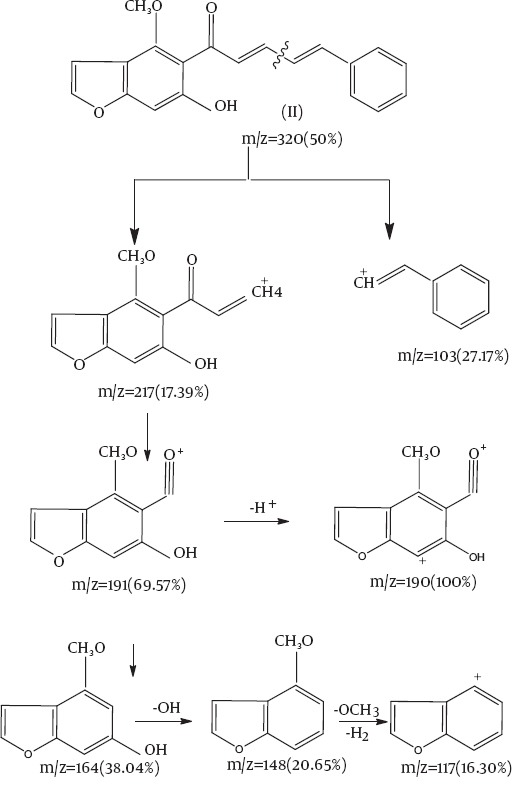


Scheme 1 outlines the synthesis of benzofuran derivative. 1-[6-hydroxy-4-methoxy benzofuran-5-yl] ethanone (**I**) reacted with cinnamaldehyde forming 1-[6-hydroxyl-4-methoxybenzofuran-5-yl]-5-phenylpenta-2,4-diene-1-one(**II**) (Scheme 1). Compound (**II**) was established by correct analytical and spectral data. The mass spectrum afforded a molecular ion peak at m/z 320 [M^+^, 50%] with a base peak at 190 and the following observed peaks at 227 (20.2%), 230 (16.3%), 164 (38.04%), 148 (20.6%) and 117 (1603%) which were compatible with the molecular formula C_20_H_16_O_4_ (Chart 1).

Furthermore, the reaction of compound (**II**) with a binucleophilic reagent was investigated. Interaction of compound (**II**) with guanidine hydrochloride in the presence of potassium hydroxide resulted in a pyrimidine derivative or its possible isomer (**IIIa-IIIb**) (scheme 1). The reaction continued via Michael addition followed by intermolecular cyclization followed by water elimination. IR spectrum of (**III**) showed the disappearance of the carbonyl group which was found in the parent compound and showed bands at 3124 & 3164 for NH_2_ and 3413 for the OH groups. Radiochemical purity of ^125^I-ASPMBF

**Figure 1:**
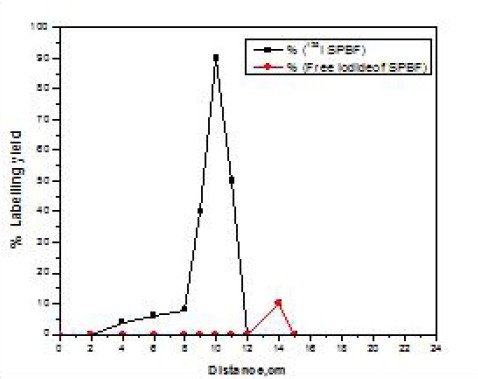
Electrophoresis radiochromatogram of ^125^I-ASPMBF

The radiochemical purity of the ^125^I-ASPMBF was determined using paper chromatography where radioiodide (^-^I) remained near the origin (R_f_ = 0 - 0.1), while the ^125^I-ASPMBF moved to the solvent front (R_f_=0.9). Radiochemical purity was further confirmed by paper electrophoresis where the radioiodide, ^125^I-ASPMBF moved to different distances away from the spotting point towards the cathode depending on the molecular weight of each one (distance from spotting point=12, 10 and 8 cm, respectively) as in (Figure 1).

### Effect of the oxidizing agents

The study was carried out to optimize the synthesis of ^125^I-ASPMBF using different oxidizing agents, including chloramines-T and iodogen. Chloramine-T (CAT) was used in the range of 1-7 nmol, and the labeling yield increased by rising the CAT concentration. A labeling yield of 90± 1% was obtained at 7 nmol of CAT as shown in table 2. Also, higher concentration of iodogen did not produce a high yield of ^125^I-ASPMBF and it took a long time (20 min) to obtain 75± 0.5% yield of ^125^I-ASPMBF with iodogen

**Table 2:**
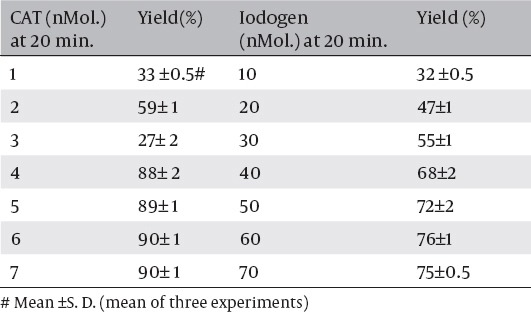
Radiochemical yield from labeling of ^125^I-ASPMBF with different concentrations of oxidizing agents

### Effect of pH

**Figure 2:**
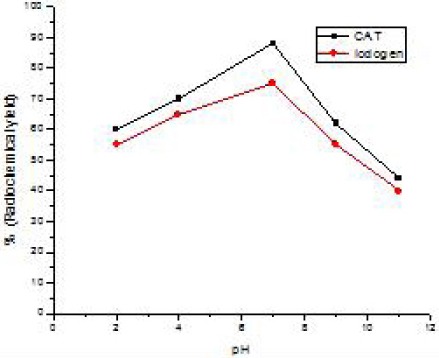
Radiochemical yield of ^125^I-SPBF as a function of pH Reaction condition: 0.2 mg of ASPMBF +10 µl Na125I (3.7 MBq) + 20 µl oxidizing agent at different pH values, reaction time was 20 min at room temperature

The hydrogen ion concentration of the reaction mixture was found to be critical. The effect of the pH on the labeling of SPBF was investigated with pH ranging from 1-10 for both oxidizing reagents (Figure 2). A yield of 90±1 was obtained at pH value equal to 7 when CAT was used as an oxidizing agent. This is due to the fact that cleavage of the aryl compounds is electrophilic in nature and is accelerated by acid. This finding is in complete agreement with the statement by El-Zahar et al (29).

### Effect of reaction time

**Figure 3:**
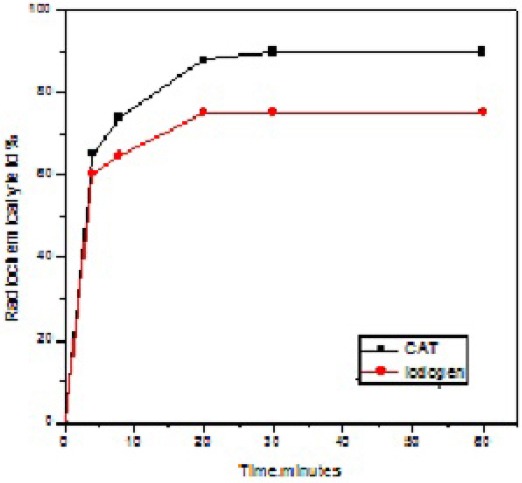
Radiochemical yield of ^125^I-SPBF as a function of reaction time. Reaction condition: 0.2 mg of substrate +10 µl Na125I + 20 µl oxidizing agent at pH value of 7 for different time intervals at room temperature.

The labeling yield of ^125^I-ASPMBF was strongly dependent on reaction time. The time points for this experiment ranged between 10 sec to 60 min and it is clear from Fig 3 that the yield was significantly increased with increase of reaction time. The results indicate that the reaction was very fast. After 2-3 min, the radiochemical yield was the same for both oxidizing agent. At 20 min the maximum radiochemical labeling (90 ± 2.3 %), (70 ± 1.5) was obtained for Chloramines-T and iodogen, respectively.

### Effect of the amount of substrate:

**Figure 4:**
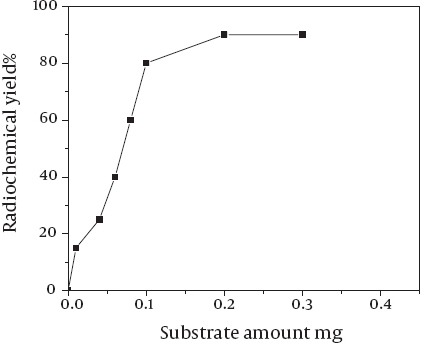
Radiochemical yield of ^125^I-SPBF as a function of substrate concentration. Reaction condition: x mg of ASPMBF +10 µl Na125I + 20 µl (CAT) at pH value of 7.5 for 20 min at room temperature.

The quantity of the substrate precursor played a role in the labeling of ASPMBF with iodine-125, but this was not as significant as the effect of pH of the reaction mixture as shown in Figure 4. Twenty micrograms of the precursor was not found sufficient to produce high yields of ^125^I-ASPMBF. Multiplying this quantity by the factor of 2.5 or 3 gives a radiochemical yield of 98 ± 1 when CAT was used as an oxidizing agent.

### The in-vitro stability

The in-vitro stability of the labeled ^125^I-ASPMBF was studied in order to determine the suitable injection for avoiding the formation of the undesired products, which resulted from the radiolysis of the labeled compounds. These undesired radioactive products might be toxic or accumulate in undesired organ. The data presented in table 3 shows that the tracer is stable for up to 5h and can be injected without any precaution, due to the formation of by products, which may be formed from the radiolysis of the labeled products.

**Table 3:**
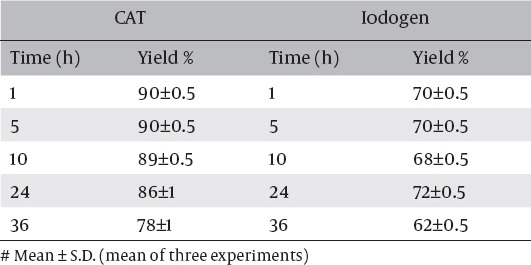
Stability of ^125^I-ASPMBF

### Biological study

The biological pathway of ^125^I was done for three healthy mice, to elucidate its normal uptake by mice organs. The data of this study are presented in tables 4 and 5. The thyroid gland is the normal trapping site of iodine, as iodide combines with triiodothyronine (T_3_) changing it to tetraiodothyronine (T_4_). Our data showed that the activity of the thyroid was more than 19 % at 30 min post injection. Benzofuran showed a high and fast brain uptake and a fast washout from the brain in the normal mice. The ^125^I-ASPMBF could be used as an imaging or a therapeutic agent. To follow up the biological distribution of the ^125^I-ASPMBF, it was injected in normal albino mice via the tail vein, and the organs uptake was determined at different time intervals. The results of this study is summarized in table 4, the ^125^I-SPBF tracer shows early high uptake in the stomach and heart equal to 6.2 % ± 0.2 and 6.9 % ± 1.3 at 5 min post injection, respectively.

**Table 4:**
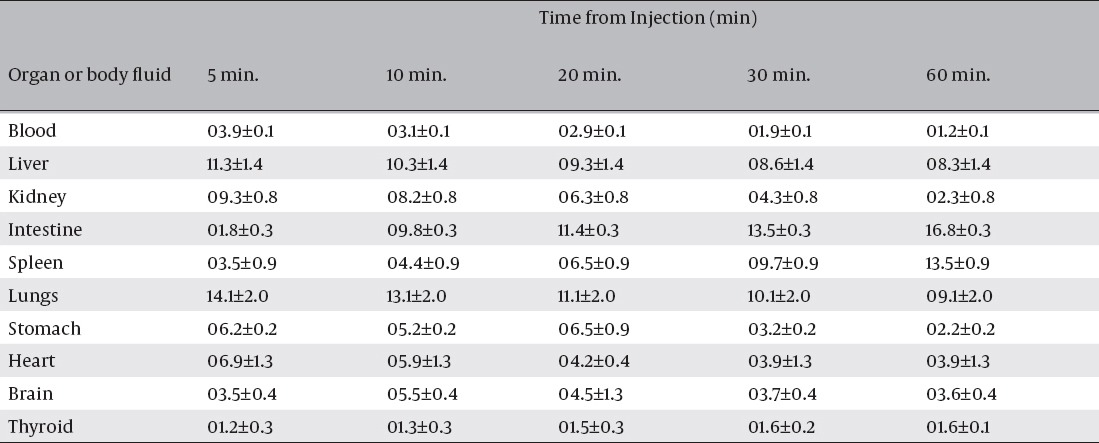
Bio-distribution of radioactivity after intravenous administration of ^125^I-ASPMBF in mice. (% ID/g ± SD, n=5)

**Table 5:**
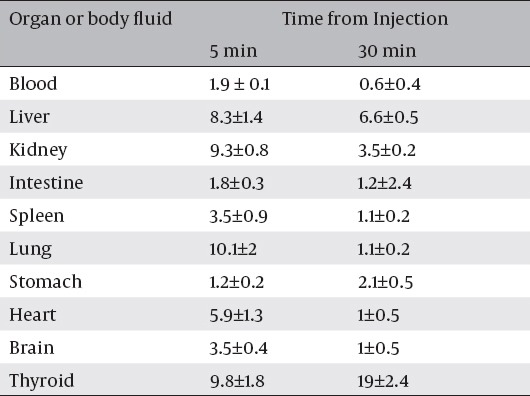
Bio-distribution of iodine-125 in normal mice (Vial content: 100 µl). (% ID/g ± SD)
